# Simultaneous determination of linezolid, meropenem and theophylline in plasma

**DOI:** 10.1016/j.dib.2018.09.097

**Published:** 2018-10-03

**Authors:** Ali K. Attia, Medhat A. Al-Ghobashy, Ghada M. El-Sayed, Samah M. Kamal

**Affiliations:** a National Organization for Drug Control and Research, Cairo, Egypt; b Analytical Chemistry Department, Faculty of Pharmacy, Cairo University, Cairo, Egypt; c Bioanalysis Research Group, School of Pharmacy, Newgiza University, Egypt

**Keywords:** Voltammetry, Determination, Linezolid, Meropenem, Theophylline, Plasma

## Abstract

The data presented in this article are related to the research article entitled “Voltammeric monitoring of linezolid, meropenem and theophylline in plasma” (A.K. Attia, M.A. Al-Ghobashy, G.M. El-Sayed, S.M. Kamal, accepted in Anal. Biochem. 2018). This article describes a sensitive square wave voltammetric (SWV) method for simultaneous monitoring of linezolid (LIN), meropenem (MERO) and theophylline (THEO) in spiked plasma and in plasma of healthy volunteers.

**Specifications table**

**Table:** 

**Subject area**	*Chemistry*
**More specific subject area**	*Electrochemical methods*
**Type of data**	*Tables, figures, text file*
**How data was acquired**	*Survey, Electrochemical workstation (SP-150, Biologic Science Instruments, France) with electrochemistry software (EC lab).*
**Data format**	*Raw, plasma, analyzed*
**Experimental factors**	*Experimental parameters such as pH, percentage of MWCNTs, and pre-concentration time were optimized*
**Experimental features**	*Preparation of working electrode, Assignment of the optimum conditions for the determination of linezolid, meropenem and theophylline in plasma*
**Data source location**	*Cairo, Egypt*
**Data accessibility**	*The Data are available with this article*

**Value of the data**

• The data presents the optimum conditions for the determination of LIN, MERO and THEO.
• This work allows to simultaneous determination of LIN, MERO and THEO in spiked plasma.
• Monitoring of LIN, MERO and THEO in real plasma samples is available.

## 1 Data

**Fig. 1:**
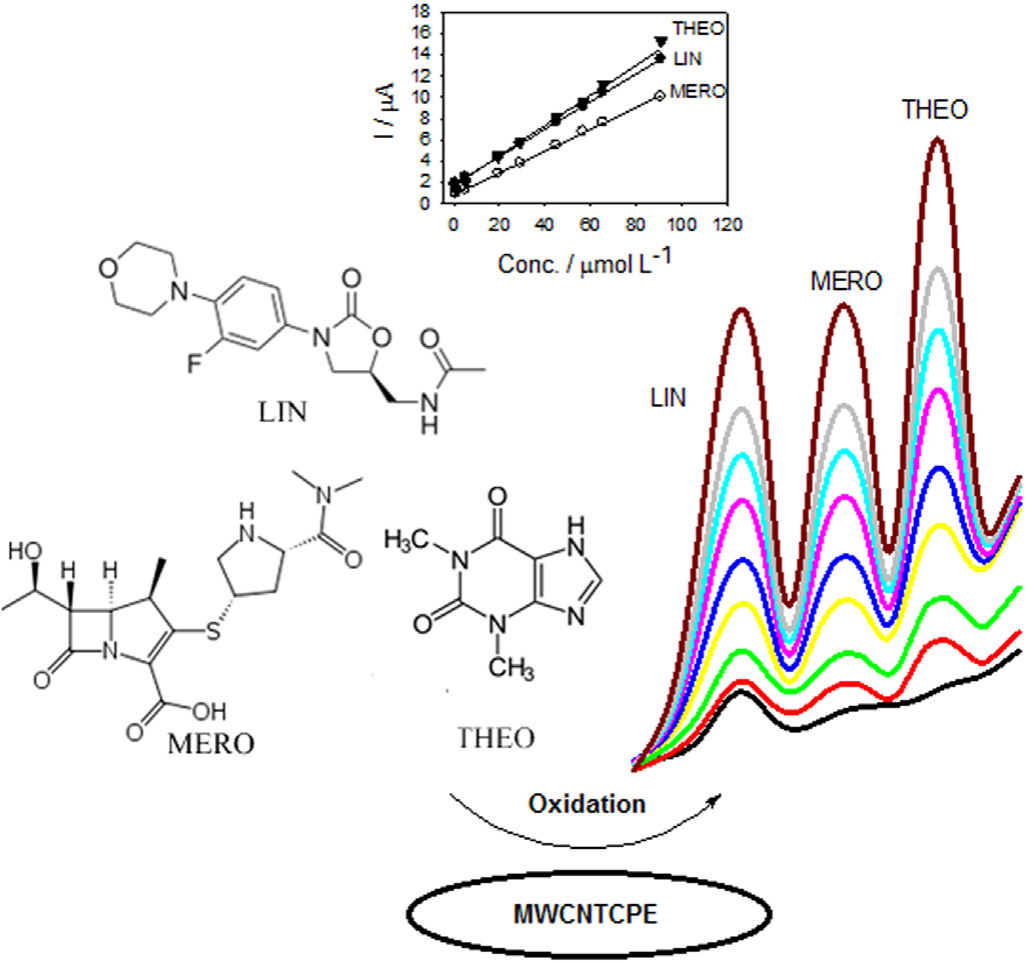
Determination of LIN, MERO and THEO in spiked plasma at MWCNTCPE in BR buffer of pH 3.0. A scan rate of 50 mV s^-1^. Inset: linear calibration curves of LIN, MERO and THEO.

The dataset of this article provides information on the determination of LIN, MERO and THEO in spiked plasma and in plasma of healthy volunteers. Fig. 1 shows the SWV voltammograms and calibration curves of the studied drugs in spiked plasma. The results are listed in Table 1. Table 2 shows the obtained concentrations of LIN, MERO and THEO in real plasma sample.

**Table 1:** Determination of LIN, MERO, and THEO in spiked plasma.

**Drug**	**Regression equation**	***R***^**2**^	**Concentration (mol L**^**-1**^**)**
LIN	*I* (µA) = 1.8459 + 0.1296 C (µmol L^-1^)	0.9998	4.0 × 10^-7^ − 9.0 × 10^-5^
MERO	*I* (µA) = 0.8509 + 0.1025 C (µmol L^-1^)	0.9994	8.0 × 10^-7^ − 9.0 × 10^-5^
THEO	*I* (µA) = 1.2984 + 0.1518 C (µmol L^-1^)	0.9984	8.0 × 10^-7^ − 9.0 × 10^-5^

**Table 2:** Determination of LIN, MERO, and THEO in real plasma sample using standard addition method.

**Drug**	**Concentration (mol L**^**1**^**)**
LIN	3.58 × 10^-5^
MERO	2.83 × 10^-5^
THEO	3.41 × 10^-5^

## 2 Experimental design, materials and methods

### 2.1 Preparation of modified electrode

The modified electrode was then prepared by adding 3.0% MWCNTs (15 mg) to graphite powder (485 mg) in ether till homogeneity was obtained. The mixture was sonicated and the ether was allowed to evaporate, and then paraffin oil (nearly 0.3 mL) was added to obtain the paste.

The area of working electrode (MWCNTCPE) was obtained using Randles-Sevcik equation: *I*_p_ = (2.69 × 10^5^) *n*^3/2^ *A C*_o_^*^ *D*_o_^1/2^ *υ*^1/2^, where Ip is the anodic peak current (A), *D*_o_ is the diffusion coefficient (7.6 × 10^-6^ cm^2^ s^-1^), υ is the scan rate (V s^-1^), n is the number of electrons exchanged during electrode reaction (*n* = 1), and *C*_o_^*^ is the concentration of K_3_Fe (CN)_6_ (1.0 × 10^-3^ mol L^-1^ K_3_Fe (CN)_6_ in 0.1 mol L^-1^ KCl). A was calculated to be 0.896 cm^2^ [1].

### 2.2 Determination of LIN, MERO, and THEO in spiked plasma

Different volumes of standard LIN solution LIN, MERO and THEO (1.0 × 10^-3^ mol L^-1^, each) were added to 10.0 mL centrifuge tubes containing 1.0 mL plasma and 3.0 mL acetonitrile, then the mixture was centrifuged and the supernatant was transferred into 5.0 mL glass vials. Aliquots of 0.5 mL of the supernatant were added to 4.5 mL of Britton-Robinson (BR) buffer of pH 3.0. SWV determinations were carried out (Pulse width of 50 ms, pulse height of 25 mV, and a scan rate of 50 mV s^-1^).

### 2.3 Determination of LIN, MERO, and THEO in real plasma

The study protocol was approved by the ethical committee of the Faculty of Pharmacy, Cairo University. All experiments were conducted as per the International Clinical Research guidelines, expressed in the Declaration of Helsinki, 1964 and revised in Brazil, 2013 [2]. Blood samples were obtained after 60 min from dose administration into 3.0 mL heparinized tubes. LIN, MERO, and THEO were determined in incurred samples using standard addition method. International Conference on Harmonization (ICH) Tripartite [3] guidelines were used to validate the proposed method.

## Figures and Tables

**Fig. S1:**
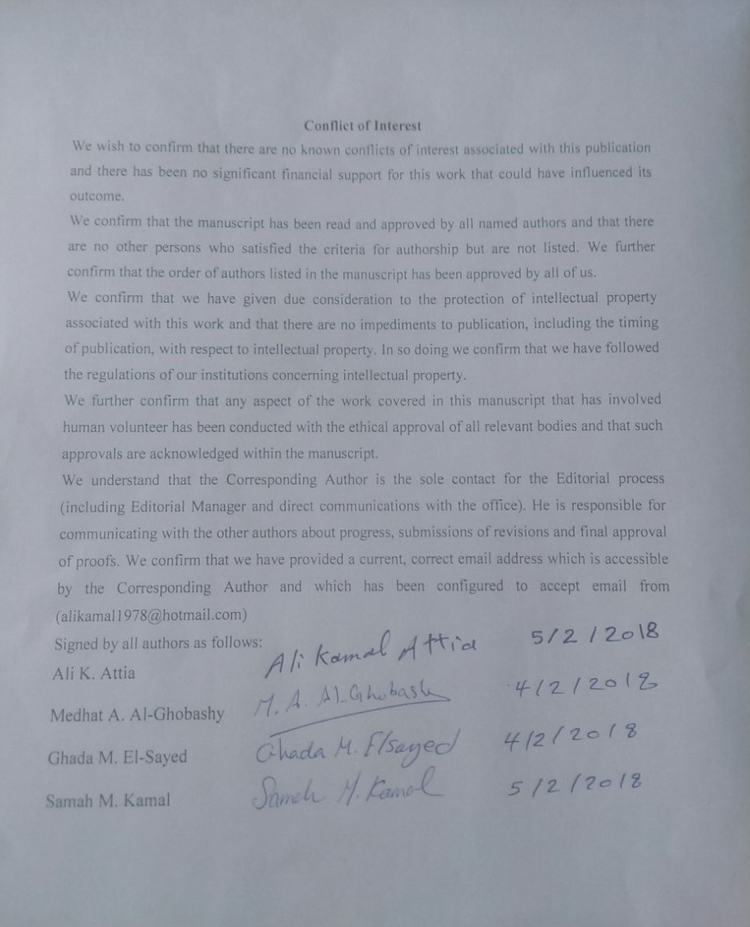
.
